# Magnetic field-induced non-linear transport in HfTe_5_

**DOI:** 10.1093/nsr/nwab208

**Published:** 2021-11-26

**Authors:** Cheng Zhang, Jinshan Yang, Zhongbo Yan, Xiang Yuan, Yanwen Liu, Minhao Zhao, Alexey Suslov, Jinglei Zhang, Li Pi, Zhong Wang, Faxian Xiu

**Affiliations:** State Key Laboratory of Surface Physics and Institute for Nanoelectronic Devices and Quantum Computing, Fudan University, Shanghai 200433, China; State Key Laboratory of Surface Physics and Department of Physics, Fudan University, Shanghai 200433, China; Zhangjiang Fudan International Innovation Center, Fudan University, Shanghai 201210, China; State Key Laboratory of High-Performance Ceramics and Superfine Microstructure, Shanghai Institute of Ceramics, Chinese Academy of Sciences, Shanghai 200050, China; School of Physics, Sun Yat-Sen University, Guangzhou 510275, China; State Key Laboratory of Precision Spectroscopy, East China Normal University, Shanghai 200062, China; State Key Laboratory of Surface Physics and Department of Physics, Fudan University, Shanghai 200433, China; State Key Laboratory of Surface Physics and Department of Physics, Fudan University, Shanghai 200433, China; National High Magnetic Field Laboratory, Tallahassee, FL 32310, USA; Anhui Province Key Laboratory of Condensed Matter Physics at Extreme Conditions, High Magnetic Field Laboratory of the Chinese Academy of Sciences, Hefei 230031, China; Anhui Province Key Laboratory of Condensed Matter Physics at Extreme Conditions, High Magnetic Field Laboratory of the Chinese Academy of Sciences, Hefei 230031, China; Institute for Advanced Study, Tsinghua University, Beijing 100084, China; Collaborative Innovation Center of Quantum Matter, Beijing 100871, China; State Key Laboratory of Surface Physics and Institute for Nanoelectronic Devices and Quantum Computing, Fudan University, Shanghai 200433, China; Shanghai Qi Zhi Institute, Shanghai 200232, China; State Key Laboratory of Surface Physics and Department of Physics, Fudan University, Shanghai 200433, China; Zhangjiang Fudan International Innovation Center, Fudan University, Shanghai 201210, China; Shanghai Research Center for Quantum Sciences, Shanghai 201315, China

**Keywords:** topological state, quantum transport, electron correlation, density wave, non-linear transport

## Abstract

The interplay of electron correlations and topological phases gives rise to various exotic phenomena including fractionalization, excitonic instability and axionic excitation. Recently discovered transition-metal pentatellurides can reach the ultra-quantum limit in low magnetic fields and serve as good candidates for achieving such a combination. Here, we report evidence of density wave and metal-insulator transition in HfTe_5_ induced by intense magnetic fields. Using the non-linear transport technique, we detect a distinct non-linear conduction behavior in the longitudinal resistivity within the *a–c* plane, corresponding to the formation of a density wave induced by magnetic fields. In high fields, the onset of non-linear conduction in the Hall resistivity indicates an impurity-pinned magnetic freeze-out as the possible origin of the insulating behavior. These frozen electrons can be gradually reactivated into mobile states above a threshold of electric field. This experimental evidence calls for further investigation into the underlying mechanism of the bulk quantum Hall effect and field-induced phase transitions in pentatellurides.

## Teaser

The nonlinear conduction is induced by magnetic fields in HfTe_5_, suggesting the formation of the density wave and an impurity-pinned magnetic freeze-out state.

## INTRODUCTION

Topological phases of matter represent a wide range of electronic systems with non-trivial band topology. New quasiparticles, such as Dirac and Weyl fermions, formed by band crossing points in these topological matters, have been discovered in experiments [1–5]. To date, most topological materials discovered fall into the scope of a single-particle picture [6,7]. Owing to the vanishing density of states in the vicinity of band crossing points, the Coulomb interaction between these Dirac/Weyl fermions becomes unscreened with a long-range component, which may present distinct correlation effects [8]. One way to enhance the correlation is by using high magnetic fields to compress dilute electrons into highly degenerate states. By considering different interactions, various scenarios were proposed theoretically [9–15] in interacting topological semimetals. An important research direction of interacting topological semimetals is to generate axionic dynamics by inducing density wave (DW) states [9]. This forms the quasiparticle of axions [16], one of the most promising candidates for dark matter. The pursuit of these goals in experiments requires a low Fermi level so that electrons can be condensed into low-index Landau levels within the accessible field range.

Layered transition-metal pentatellurides ZrTe_5_ and HfTe_5_ have recently been found to be close to an accidental Dirac semimetal phase in the boundary between strong and weak topological insulators, sensitively affected by the lattice constant [17–22]. While the exact value of the band gap is under debate, the bulk states of these systems can be regarded as Dirac fermions with a small mass term [19,23,24]. Angle-dependent quantum oscillations in ZrTe_5_ revealed a tiny Fermi surface, with a rod-like ellipsoid shape and large anisotropy in the Fermi wave vector and effective mass between the out-of-plane direction (*b* axis) and the in-plane directions (*a* and *c* axes) [20]. Owing to easy access to the quantum limit within a moderate field, the chiral anomaly [18,25,26], log-periodic quantum oscillations [27–29], the anomalous Hall/Nernst effect [22,30–32] and quantized plateaus of Hall resistivity [33–35] have been observed in bulk crystals and flakes of ZrTe_5_ as well as its cousin HfTe_5_. These observations suggest that pentatellurides are good candidates of topological systems for studying the electron correlation effect in magnetic fields.

## RESULTS

**Figure 1.**
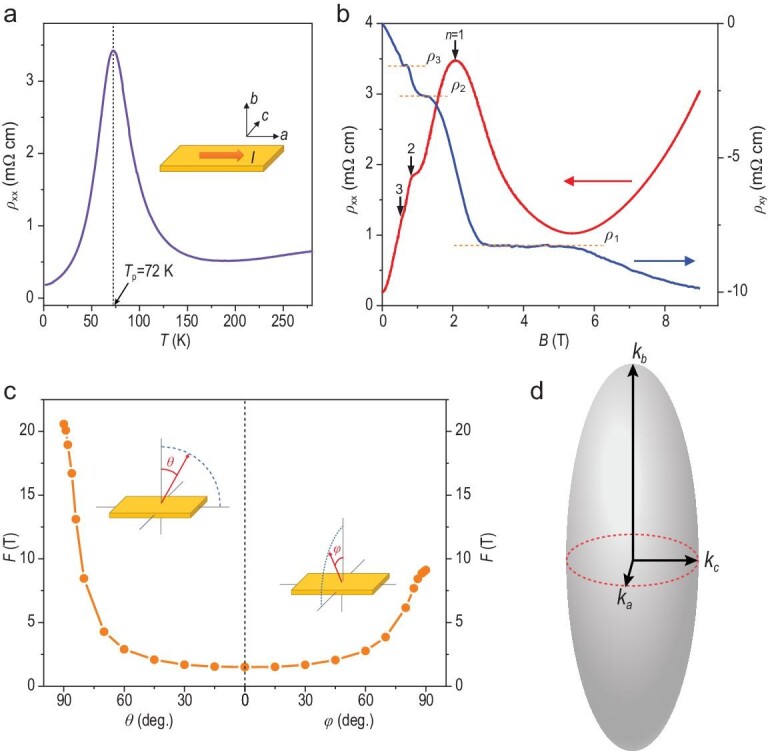
Magneto-transport and Fermi surface anisotropy of HfTe_5_. (a) The zero-field resistivity of HfTe_5_ as a function of *T* with a peak at 72 K. The current is applied along the *a* direction within the *a–c* plane. (b) The longitudinal (*ρ_xx_*, the red curve) and Hall (*ρ_xy_*, the blue curve) resistivity at 2 K with *n* the Landau level index. Note that the original longitudinal and Hall resistance data of this figure (without normalization with sample geometry) have been presented in Fig. S18d of ref. [36] by some of the authors. The dashed lines mark the position of Hall plateaus. (c) The oscillation frequencies as a function of *θ* and *ϕ*. The insets are the measurement geometries. (d) A sketch of the Fermi surface in HfTe_5_.

In this study, by combining linear and non-linear quantum transport, we present evidence of field-induced DW and insulating states in HfTe_5_, which can be further modulated by an electrical field bias. Single crystals of HfTe_5_ were produced by iodine-assisted chemical vapor transport (CVT), as described in Section I of the supplementary materials. HfTe_5_ has a carrier density of 2.7 × 10^17^ cm^–3^ at 2 K, one order of magnitude lower than that of ZrTe_5_ (2.5 × 10^18^ cm^–3^) grown by the same method [36]. The low carrier density enables easy access to the quantum limit. We performed transport experiments with the current along the *a* axis and the magnetic field along the *b* axis was noted as a *θ* = 0° configuration. Figure 1a shows the temperature-dependent resistivity with a peak around 72 K in Sample H1. This peak comes from the shift of Fermi energy from the valence band towards the conduction band as the temperature decreases. A similar transition also occurs in CVT-grown ZrTe_5_, as indicated by the sign change in both Hall and Seebeck coefficients with temperature [37]. Figure 1b shows the longitudinal magnetoresistivity (*ρ_xx_*, the red curve) and Hall resistivity (*ρ_xy_*, the blue curve) with clear oscillations at *θ* = 0°. Apart from small quantum oscillations at low fields, *ρ_xx_* experiences a large peak around 2 T when *θ* = 0°, followed by a dip at 5.4 T. As marked by the Landau level index *n* at resistivity peaks, HfTe_5_ enters the quantum limit around 2 T, where only the lowest zeroth Landau levels are occupied. The oscillation peak at *n *= 1 reaches a much larger magnitude compared to the others at lower fields. Meanwhile, *ρ_xy_* shows a series of plateau-like features as a function of *B*, in coordination with the oscillations in *ρ_xx_*. It resembles the bulk quantum Hall (QH) effect recently observed in ZrTe_5_ [33,38,39] and HfTe_5_ [34,35] but with a finite longitudinal resistivity residue. By tracking the angle dependence of quantum oscillations within the *a-b* and *c-b* planes (Fig. S1a and b), we can extract oscillation frequencies as functions of angles, as shown in Fig. 1c (*θ* for the *a–b* plane and *ϕ* for the *c–b* plane, as illustrated in the insets of Fig. 1c). It suggests that the Fermi surface of HfTe_5_ also adopts an ellipsoid shape (Fig. 1d) similar to that of ZrTe_5_. The fitting of the oscillation frequencies in Fig. 1c yields a strong anisotropy as *k_a_*: *k_b_*: *k_c__ _*= 1 : 13.6 : 2.3, with *k_a_*, *k_b_* and *k_c_*, the lengths of the three semi-axes in the Fermi ellipsoid, being 0.0045, 0.0613 and 0.0102 Å^−1^, respectively.

**Figure 2.**
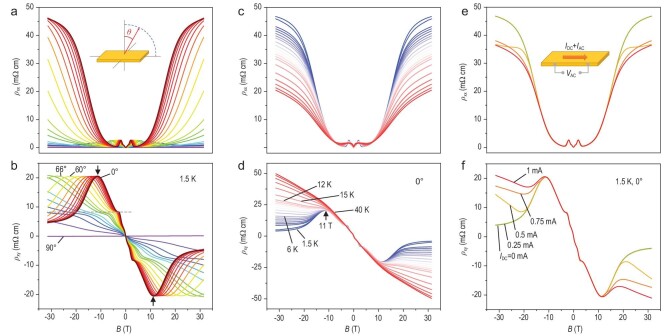
Linear and non-linear transport results of HfTe_5_ at high magnetic fields. (a and b) *ρ_xx_* (a) and *ρ_xy_* (b) as a function of *B* at different angles at 1.5 K. The inset of (a) is the measurement geometry. The intervals between curves in the ranges *θ* = 0°– 60° and *θ* = 66°–90° are 6° and 3°, respectively. The transition in Hall effect is marked by the black arrows. (c and d) *ρ_xx_* (c) and *ρ_xy_* (d) at different temperatures at *θ* = 0°. The intervals between curves in the ranges 1.5–6 K, 6–12 K and 15–40 K are 0.5 K, 1 K and 5 K, respectively. The crossing point of *ρ_xx_* and the transition in *ρ_xy_* are marked by the black arrows. (e and f) *ρ_xx_* (e) and *ρ_xy_* (f) at different DC currents at *θ* = 0° and 1.5 K. The inset of (e) is the measurement configuration. Each color represents the same *θ*, temperature and current for (a) and (b), (c) and (d), and (e) and (f), respectively. Note that the field-dependent magnetoresistivity data in this figure have been symmetrized (or anti-symmetrized for Hall effect) in *B* with the original data shown in Fig. S4.

We further investigated the transport properties of HfTe_5_ in higher magnetic fields. Figure 2a and b show *ρ_xx_* and *ρ_xy_* of another HfTe_5_ crystal (Sample H2) measured in a resistive magnet up to 31.5 T at different angles. For *θ* = 0°, the magnetoresistivity in the low-field regime reproduces the results presented in Fig. 1 well, with a large dip in *ρ_xx_* at ∼4 T. Subsequently, *ρ_xx_* continues increasing and gradually saturates above 21 T. No further oscillations appear since only the lowest Landau level is occupied. Notably, *ρ_xy_* presents an anomalous peak-like feature (marked by the black arrows in Fig. 2b) at 11 T, then starts to decrease and finally saturates above ∼21 T. As the field is tilted toward the in-plane direction, the magnetoresistivity ratio gets suppressed and the peak position (*B*_p_) in *ρ_xy_* moves towards higher magnetic fields. In Fig. S1d, we show that *B*_p_ can be fitted by the cosine function of the tilting angle *θ*, suggesting that the peak of *ρ_xy_* is likely to be induced by the out-of-plane component of the magnetic field. Meanwhile, the plateau at *ρ_xy_* = *ρ*_1_ persists at large *θ* as marked by the gray dashed line in Fig. 2b. The temperature dependencies of *ρ_xx_* and *ρ_xy_* are plotted in Fig. 2c and d, respectively. The suppression of *ρ_xy_* in the high-field regime quickly disappears above 15 K and *ρ_xx_* decreases drastically, while the low-field parts of *ρ_xy_* overlap well at different temperatures. The field dependence of *ρ_xx_* at 1.5–6 K shows a crossing point around 11.8 T (Fig. S6). It corresponds to a field-induced metal-insulator transition, which follows a scaling relation with *T* and *B* in the vicinity of the crossing point as shown in Section III of the supplementary materials. The strong temperature dependence (1.5–15 K) of the high-field parts in *ρ_xx_* and *ρ_xy_* suggests that the peak in *ρ_xy_* is unlikely to originate from the trivial multi-carrier transport mechanism. By considering the field dependence of both *ρ_xx_* and *ρ_xy_*, we conclude that HfTe_5_ enters an insulating state around 11.8 T, where *ρ_xx_* is enhanced and *ρ_xy_* is suppressed due to the decrease in the number of mobile carriers.

Strong non-linear transport of HfTe_5_ was observed in magnetic fields. We started by applying a series of direct currents (DC) (0–1 mA) with a superimposed alternating current (AC) of 50 μA to the sample and detected the AC resistance using the standard lock-in technique. As presented in Fig. 2e and f, the curves overlap for *I*_DC _= 0 and 0.25 mA. As *I*_DC_ exceeds 0.5 mA, the suppression of *ρ_xy_* at high fields is gradually eliminated and *ρ_xx_* decreases as well in the same regime. The bias-dependent resistivity indicates that the insulating phase of HfTe_5_ at high fields is suppressed upon large biases.

**Figure 3.**
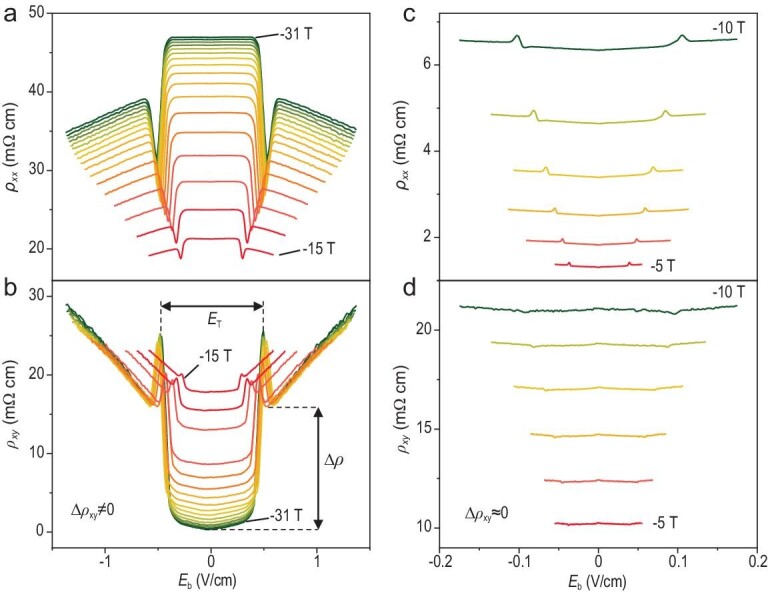
Bias-dependence of resistivity tensors at different magnetic fields. (a and b) *ρ_xx_* (a) and *ρ_xy_* (b) as a function of *E*_b_ fields at 1.5 K. The interval between curves is 1 T. (c and d) *ρ_xx_* (c) and *ρ_xy_* (d) as a function of *E*_b_ at 1.5 K. The interval between curves is 1 T. The value of *E*_b _= 1 V/cm corresponds to a DC current of 1.09 mA at 31 T.

To track the evolution of the non-linear behavior, we investigated the bias-dependent DC differential resistivity. In Fig. 3a and b, the differential resistivity (*ρ_xx_* and *ρ_xy_*) is plotted as a function of the biased electric field *E*_b_ above −15 T. Under small biases, the system remains in the linear transport regime with differential resistivity values fixed at different biases. Sharp transitions from linear to non-linear regimes occur above a threshold electric field, *E*_T_. In the range of −15 to −31 T, *ρ_xx_* (*ρ_xy_*) shows a prominent dip (peak) at *E*_T_ followed by a linear decrease (increase) upon the increase of *E*_b_. In contrast, in the range of −5 to −10 T, *ρ_xx_* increases with *E*_b_ beyond the threshold, while *ρ_xy_* becomes nearly independent of *E*_b_ except for a slight variation near *E*_T_. It is in stark contrast to the conventional joule heating effect induced by large currents. The suppression of in-plane transport is gradually relieved by increasing *E*_b_ and the carriers become mobile again. The sharp transition near the onset of the non-linearity will smear out as the temperature increases but the non-linear transport persists (Fig. S5c). One unexpected finding is that *ρ_xx_* continues to show non-linear transport in the range of 0 to −10 T (where the linear transport shows no anomaly) while *ρ_xy_* does not (Fig. 3c and d and Fig. S5a). At zero magnetic field, the non-linear behavior vanishes (i.e. the resistivity becomes independent of the applied biases).

**Figure 4.**
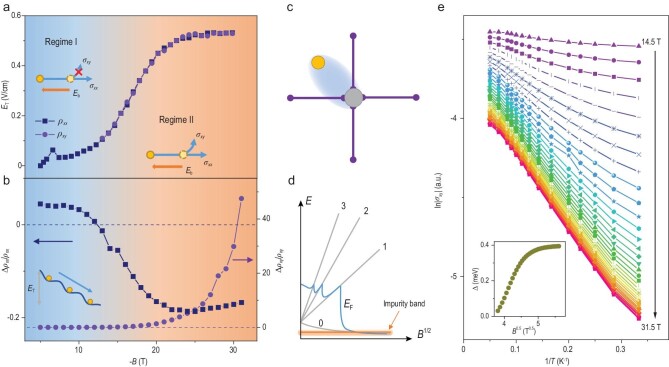
The DW and insulating phases of HfTe_5_ in magnetic fields. (a and b) *E*_T_ (a) and Δ*ρ*/*ρ* (b) as a function of *B*. The navy blue and purple dots correspond to *ρ_xx_* and *ρ_xy_*, respectively. Δ*ρ*/*ρ* is the resistivity difference Δ*ρ* before and after the transition (Fig. 3b) divided by the zero-*E*_b_ value of the resistivity. The insets in (a) show the non-linear behavior in *ρ_xy_*, which separates the phase diagram of HfTe_5_ into two regimes. In Regime I, only the longitudinal transport will be activated. In Regime II, both longitudinal and Hall transports will be activated when the electric field breaks the binding between electrons and impurities. The inset in (b) is a schematic of the DW electron sliding. (c) The magnetic freeze-out state where the carriers bind to the local impurities (the gray circle). (d) The schematic band structure for the magnetic freeze-out effect. At high fields the itinerant electrons are pinned to the impurity band. (e) Arrhenius plots of |*σ_xy_*|. The inset is the activation gap as a function of *B*^0.5^.

In Fig. 4a and b, we extract the threshold electric field *E*_T_ and the relative resistivity change Δ*ρ*/*ρ* near the transition as a function of magnetic fields. The relative resistivity change quantifies the effect on the transport property. The difference in non-linear conduction of diagonal and off-diagonal components in resistivity tensors separates the phase diagram of HfTe_5_ under magnetic fields into two regimes. In Regime I, HfTe_5_ remains metallic and only diagonal resistivity from longitudinal transport is affected by bias fields (top-left inset in Fig. 4a). This behavior is consistent with the widely studied sliding motion of DW states. A bias exceeding *E*_T_ results in a 1D axial motion and does not contribute to Hall effect [40,41]. The differential resistivity curves shown in Fig. 3 are symmetric with electric fields and change linearly after the depinning transition, while other origins of non-linear resistivity such as hopping conduction or *p–n* junctions give dramatically different behaviors (activation behavior in electric fields for hopping conduction [42], non-symmetric I–V curve for *p–n* junctions or other interface potential barriers [43]). The sharp dip/peak features near the transition and the threshold electric field value are consistent with the typical sliding motion of density wave systems [44–46]. In Regime II, electrons in HfTe_5_ become strongly localized and a large bias can activate both diagonal and off-diagonal resistivity tensors, making the system metallic again. Such behavior is distinct from the sliding DW case and fits the depinning process of defect-localized electron solid states. A large bias breaks the binding between electrons and impurities and makes electrons mobile again. Then both longitudinal and Hall transport (*ρ_xx_* and *ρ_xy_*) will be activated (bottom-right inset in Fig. 4a). We note that although a classical picture involving the ‘backflow’ of normal electrons can affect Hall resistivity in the sliding DW state [47], it leads to a decrease rather than an increase of |*ρ_xy_*| and cannot give such a large change in Δ*ρ_xy_*/*ρ_xy_*, contradictory to our observation.

## DISCUSSION

In a previous study [33], the emergence of bulk QH effect in ZrTe_5_ was interpreted as a DW forming along the *b* direction induced by Landau level nesting. Similar to ZrTe_5_, HfTe_5_ shows signatures of QH effect [34,35], which indicates that the interlayer dispersion is diminished. Nevertheless, the Landau level nesting picture cannot account for the in-plane non-linear transport since it only induces a DW along the *b* direction. The absence of the sliding behavior at zero field and the systematic increase of *E*_T_ with *B* indicate that the DW state is induced by magnetic fields. The picture of a field-induced spin DW, as observed in organic conductors [48], matches our results in a better way, while the charge DW order will normally be suppressed by the magnetic fields [49]. According to the quantized nesting model [48], the strength of a DW is governed by the Fermi surface anisotropy (refer to Section IV of the supplementary materials for detailed discussions). Therefore, the interlayer periodic potential confines the system into a series of 2D planes, leading to the emergence of the bulk QH effect. Above 2 T, the carriers are confined to the zeroth Landau levels. The in-plane transport can be evaluated via the bias-dependent differential resistivity measurements. Only few carriers are localized by the DW at low fields as evidenced by the relatively small change of the resistivity between the pinning and sliding states. This conclusion also explains another unusual behavior—the sign of Δ*ρ_xx_*/*ρ_xx_* oscillating with quantum oscillations at low fields shown in Fig. S5a. Both the normal and DW electrons coexist in the system with the former being the majority. Under large biases beyond *E*_T_, the DW electrons start a 1D sliding motion and contribute to the conduction. Then, according to the two-fluid model, the current of normal electrons becomes smaller since the total current is fixed. Therefore, the quantum oscillations given by normal electron conduction will become comparably weaker, which results in the oscillating sign of Δ*ρ_xx_*/*ρ_xx_* with *B* as shown in Fig. S5a.

Based on these observations, we propose a possible scenario for the phase diagram of HfTe_5_ in magnetic fields. The gap size of HfTe_5_ decreases with temperature, resulting in the low-temperature phase close to an accidental Dirac semimetal [19,21]. At zero field, the Fermi surface of HfTe_5_ resembles a highly anisotropic ellipsoid filled with electrons. With magnetic fields along the *b* direction, the system is firstly driven to a 3D DW state, then gradually collapses into an impurity-pinned insulating state around 11.8 T. Generally, there are mainly two kinds of mechanisms accounting for the defect-pinned insulating phase in the quantum limit of dilute electronic systems. One is the formation of Wigner crystals [50]. It is a collective state where the potential energy of electrons dominates the kinetic energy and the electrons crystallize into a lattice. However, the typical temperature required to form a Wigner crystal is in the milli-Kelvin range while the insulating phase in HfTe_5_ persists up to over 15 K. Besides, the critical field of Wigner transition was found to be temperature dependent [51] while the transition features in HfTe_5_ were found to be temperature insensitive. The other mechanism is the magnetic freeze-out effect, which originates from the electron-impurity interaction [52]. In this case, electrons bind to impurities due to the small magnetic length at high fields (Fig. 4c). In Section IV of the supplementary materials, a rough estimation based on the effective mass and the carrier density in HfTe_5_ gives the value field of the magnetic freeze-out *B*_c _≈ 8.2 T, close to the experimental results. In the view of band structure, the Fermi level meets the impurity band at high magnetic fields and electrons are localized near the impurity sites as illustrated in Fig. 4d. Thus, the Hall conductivity should be thermally activated by temperature as ${\sigma _{xy}} \propto {e^{ - {\varepsilon _b}/kT}}$ with ${\varepsilon _b}$ being the field-dependent binding energy and $k$ the Boltzmann constant. As shown in Fig. S6, the absolute value of the Hall conductivity increases with temperature above 14 T. Figure 4e shows the activation of ${\sigma _{xy}}$ in different fields. The fitted activation gap Δ as a function of the square root of the magnetic field is plotted in the inset of Fig. 4e. It increases linearly with $\sqrt B $ in the range of 14.5–20 T and gradually saturates to ∼0.4 meV after that, in agreement with the magnetic freeze-out case discussed in Section IV of the supplementary materials.

One interesting feature is that *ρ_xx_* only changes by <40% at different biases while *ρ_xy_* is suppressed to nearly zero in the high-field localized states. In Fig. S7, we show that in high fields the Hall component of conductivity gradually exhibits different temperature dependence from the longitudinal part. While further investigations are required, it may be related to possible 1D edge modes as proposed recently in KHgSb [53]. The observations of in-plane non-linear conduction and a possible magnetic freeze-out state indicate a complicated phase diagram of HfTe_5_ in magnetic fields. Thermodynamic properties and the *ac* transport of a sliding DW may be investigated to further identify the nature of these phase transitions. Experimental methods sensitive to spin polarization can be applied to establish whether it is a charge or spin DW. Meanwhile, the unique electromagnetic response (axion electrodynamics) in topological systems allows for the fluctuation of topological *θ*-term, which behaves like the axion and gives rise to novel topologically protected transport properties, in the presence of the DW order [9,54]. The DW state of HfTe_5_ in magnetic fields can be therefore used to explore the physics of axionic dynamics [9,12,54].

## METHODS

High-quality single crystals of HfTe_5_ were grown via CVT with iodine as the transport agent, similar to ZrTe_5_ [20]. Stoichiometric Hafnium flakes (99.98%, Aladdin) and Tellurium powder (99.999%, Alfa Aesar) were ground together and sealed in an evacuated quartz tube with iodine flakes (99.995%, Alfa Aesar). A temperature gradient of 60°C between 490°C and 430°C in a two-zone furnace was used for crystal growth. The typical as-grown sample has a long ribbon-like shape along the *a* axis. The sample crystalline quality and stoichiometry were checked by the X-ray diffraction and energy dispersive spectrum. The magneto-transport measurements were performed in a Physical Property Measurement System (Quantum Design) for the low fields and in resistive magnets in Tallahassee, USA, and Hefei, China, for the high fields with the standard lock-in technique. The differential resistivity measurements were carried out using the corresponding built-in mode of Keithley 6221 and 2182 models. The bias electric field is extracted as ${E_b} = \ IR/L$ with $I$, $R$ and $L$ being the applied current, four-terminal resistance value and channel length between two inner voltage-sensing electrodes, respectively.

## Supplementary Material

nwab208_Supplemental_File
