# Supplementary material for: Magnetic field-induced non-linear transport in HfTe5
Source: Natl Sci Rev. 2021 Nov 26;9(10):nwab208. doi: 10.1093/nsr/nwab208 (PMC9645650; doi:10.1093/nsr/nwab208)
Supplement: nwab208_Supplemental_File [file nwab208_supplemental_file.docx]

**Supplementary Materials for**

**Magnetic-field-induced nonlinear transport in HfTe_5_**

Cheng Zhang^1,3,4#^**^*^**, Jinshan Yang^5#^, Zhongbo Yan^6#^, Xiang Yuan^7^, Yanwen Liu^3^, Minhao Zhao^3^, Alexey Suslov^8^, Jinglei Zhang^9^, Li Pi^9^, Zhong Wang^10,11^, Faxian Xiu^1,2,3,4,12^**^*^**

^1^ Institute for Nanoelectronic Devices and Quantum Computing, Fudan University, Shanghai 200433, China

^2^ Shanghai Qi Zhi Institute, Shanghai, 200232, China

^3^ State Key Laboratory of Surface Physics and Department of Physics, Fudan University, Shanghai 200433, China

^4^ Zhangjiang Fudan International Innovation Center, Fudan University, Shanghai 201210, China

^5^ State Key Laboratory of High Performance Ceramics and Superfine Microstructure, Shanghai Institute of Ceramics, Chinese Academy of Sciences, Shanghai 200050, China

^6^ School of Physics, Sun Yat-Sen University, Guangzhou 510275, China

^7^ State Key Laboratory of Precision Spectroscopy, East China Normal University, Shanghai, 200062, China

^8^ National High Magnetic Field Laboratory, Tallahassee, Florida 32310, USA

^9^ Anhui Province Key Laboratory of Condensed Matter Physics at Extreme Conditions, High Magnetic Field Laboratory of the Chinese Academy of Sciences, Hefei 230031, China

^10^ Institute for Advanced Study, Tsinghua University, Beijing 100084, China

^11^ Collaborative Innovation Center of Quantum Matter, Beijing 100871, China

^12^ Shanghai Research Center for Quantum Sciences, Shanghai 201315, China

^#^ These authors contributed equally to this work

**^*^** Correspondence and requests for materials should be addressed to F. X. (E-mail: [Faxian@fudan.edu.cn](mailto:Faxian@fudan.edu.cn)) or C. Z. (E-mail: [Zhangcheng@fudan.edu.cn](mailto:Zhangcheng@fudan.edu.cn))

**Contents:**

1. **Additional transport results of HfTe_5_**
2. **Temperature and magnetic field dependences of nonlinear transport**
3. **Activation behavior of Hall conductivity in high fields**
4. **Theoretical analysis of the field-induced density wave and magnetic freeze-out states**
5. **References**
6. **Additional transport results of HfTe_5_**

Figure S1a-b shows the angle dependence of longitudinal resistivity *ρ_xx_* as a function of magnetic field for sample H1 when the field is tilted in the *b-a* and *b-c* planes. The revealed Fermi surface for the *k_b_-k_c_* plane has a smaller anisotropy than that of the *k_b_-k_a_* plane as summarized in Fig. 1c. At *θ*=90°, the quantum oscillations persist (Fig. S1c), indicating a closed anisotropic Fermi surface in HfTe_5_ rather than an open one. The turning point of Hall conductivity *ρ_xy_* of sample H2 as a function of *θ* is shown in Fig. S1d, which fits well to the *B*_p0_/cos*θ* function. It suggests that only the out-of-plane magnetic field is responsible for the suppression of Hall conductivity.

The Landau level index of each oscillation is linearly fitted with 1/*B* (the position of oscillation peaks and valleys). Then the corresponding slope yields the oscillation frequency *F*. In Fig. S1e, the Landau fan diagrams with *B* along the *a*, *b*, and *c* directions are plotted. From the equation $F={(\phi}_{0}/2\pi^{2})S_{F}$ with $\phi_{0}=h/2e$, the cross-section area of the Fermi surface can be determined. Since the Fermi surface of HfTe_5_ is an ellipsoid shape as shown in Fig. 1d, the cross-section area can be expressed as $S_{F}=\pi k_{i}k_{j}$, with $k_{i}$, $k_{j}$ being the principal axes of the ellipse. Therefore, the three Fermi wave vector can be extracted as $k_{a}=0.0045 Å^{-1}$, $k_{b}=0.0613 Å^{-1}$ and $k_{c}=0.0102 Å^{-1}$, respectively. The relative oscillation amplitude is described by $\frac{\Delta\rho\left( T \right)}{\Delta\rho\left( 2 K \right)}=\frac{\lambda(T)}{\sinh\lambda(T)}$ with $\lambda\left( T \right)=\frac{2\pi^{2}k_{B}Tm^{*}}{\hbar eB}$. Here, $k_{B}$ is the Boltzmann’s constant, $\hbar$ is the reduced Plank constant, and $m^{*}$ is the effective mass. By extracting the temperature dependence of relative oscillation amplitude (Fig. S1f), we obtain the effective mass value in three planes (a-b, b-c, and a-c) as $m_{a-b}^{*}=0.138m_{e}$, $m_{b-c}^{*}=0.184m_{e}$, and $m_{a-c}^{*}=0.020m_{e}$, respectively. $m_{e}$ denotes the electron mass. The mobility value for samples H1 and H2 at 2 K are 1.26×10^5^ cm^2^/Vs and 7.52×10^5^ cm^2^/Vs, close to the recently reported mobility in HfTe_5_^1–4^. Using the formula $\mu=\frac{e\tau}{m^{*}}$, the transport life time $\tau$ can be calculated as 1.4×10^-12^ s and 8.6×10^-12^ s, respectively. Taking the reported Fermi velocity value of HfTe_5_^4^, the electron mean free path is estimated as 0.78 and 4.8 μm, respectively.

Figure S2 illustrates the band structure of HfTe_5_. At low temperatures, the bulk HfTe_5_ is a massive Dirac system with *n*-type carriers. In magnetic fields, the zeroth Landau levels adopt half of the degeneracy of other Landau levels^5^.

To check the anisotropy of the insulating phase in high fields, we also carried out transport measurements with an excitation current applied along the *c* direction, as shown in Fig. S3. Similarly, the Hall resistivity *ρ_xy_* is suppressed above ~11 T while the longitudinal resistivity *ρ_xx_* keeps increasing with the field. It suggests that the carriers are not just confined along a certain direction but rather are strongly localized for the whole *a-c* plane. Here *ρ_xx_* along the *c* direction is much larger than the value along the *a* direction due to a large in-plane mobility anisotropy. The mobility along the *c* direction is much smaller than the one at the *a* direction, which is also verified by the relatively small magnetoresistivity ratio (Fig. S3a).

Figure S4 presents the original data for each panel in Fig. 2. There is a slight influence from other channels in the original data (a small part of $\rho_{xy}$ in $\rho_{xx}$ and vice versa) mainly due to small misalignments between electrodes. But the overall field dependence at different angles, temperatures and biases remains unchanged before and after symmetrization. The validity of the symmetrization process is based on the assumption that Onsager’s reciprocity remains being obeyed in the nonlinear regime for HfTe_5_. The nonlinear transport in HfTe_5_ comes from the transition of localized electrons into mobile states, which is not affected by the reversal of magnetic field direction.

**Figure S1. Transport results of HfTe_5_.** (**a**) *ρ_xx_* as a function of magnetic field at different angles *θ* at 2 K for sample H1. The inset is the measurement geometry. (**b**) *ρ_xx_* as a function of magnetic field at different angles at 2 K. The inset is the measurement geometry. (**c**) *ρ_xx_* as a function of magnetic field at different temperatures at *θ*=90°. (**d**) The extracted turning position of Hall conductivity *ρ_xy_* of sample H2 measured in high fields as a function of *θ*, which fits to a *B*_p0_/cos*θ* function as indicated by the dashed line. (**e**) The Landau level fan diagrams with *B* along the *a*, *b*, and *c* directions, respectively. The integer and half-integer Landau level indexes correspond to oscillation peaks and valleys, respectively. (**f**) The temperature dependence of relative oscillation amplitude with *B* along three crystal axes.

**Figure S2. The schematics of band structure and Landau levels in HfTe_5_.** The bulk states of HfTe_5_ can be considered as a massive Dirac system. Due to the finite band gap, the original Dirac point is split into two zeroth Landau levels at conduction and valance bands, each with half of the degeneracy of other Landau levels.

**Figure S3. In-plane transport results with the excitation current applied along the *c* direction.** (**a-b**) *ρ_xx_* (**a**) and *ρ_xy_* (**b**) of sample H3 as a function of magnetic field at different temperatures. The inset in **a** presents the measurement geometry. The magnetic field is applied along the *b* direction.

**Figure S4. Original transport results of HfTe_5_ (Sample H2) at high magnetic fields.** (**a-b**) *ρ_xx_* (**a**) and *ρ_xy_* (**b**) as a function of magnetic field at different angles at 1.5 K. The inset of **a** is the measurement configuration. (**c-d**) *ρ_xx_* (**c**) and *ρ_xy_* (**d**) as a function of magnetic field at different temperatures at *θ*=0°. (**e-f**) *ρ_xx_* (**e**) and *ρ_xy_* (**f**) as a function of magnetic field at different DC currents at *θ*=0° and 1.5 K. The inset of **e** is the measurement configuration. Each color represents the same angle/temperature/bias as the corresponding panel of Fig. 2 in the main text.

1. **Magnetic field and temperature dependences of nonlinear transport**

An anomalous feature of the nonlinear transport in the density wave regime (-5~-10 T, Fig. 3c) is the increase of resistivity in the sliding state. Normally, as illustrated in Fig. 4a, a large bias voltage above the threshold drives the trapped carriers into the sliding motion and reduces the resistivity as widely observed^6–8^. But the relative resistivity change shows a non-monotonic trend with the magnetic field as summarized in Fig. 4b. In Fig. S5a, we present a thorough comparison of the relative resistivity *ρ*/*ρ*_0_ and quantum oscillations at different magnetic fields. The sign of the relative resistivity (the increase or decrease of resistivity in the sliding state) is related to the quantum oscillations. The relative resistivity will be positive in the valley regime of quantum oscillations and negative in the peak regime. Both the sign and the absolute value of Δ*ρ_xx_*/*ρ_xx_* as well as the value of threshold electric field *E*_T_ coordinate with those of quantum oscillations. The resistivity change is likely to be dominated by the modulation of quantum oscillation amplitude upon the sliding transition. According to the analysis in the main text, only a small part of carriers is localized by the DW at low fields as evidenced by the relatively small change of the resistivity between the pinning and sliding states. Both the normal and DW electrons coexist in HfTe_5_ with the former being the majority and contributing the quantum oscillations. Under large biases beyond *E*_T_, the DW electrons start one-dimensionally sliding motion and contribute to the conduction. Then according to the simple two-fluid model, the current of normal electrons becomes smaller since the total current is fixed. Therefore, the quantum oscillations given by normal electron conduction will become comparably weaker, which results in the oscillating sign of Δ*ρ_xx_*/*ρ_xx_* with *B* as shown in Fig. S5a.

In Fig. S5b, we present the nonlinear transport results of HfTe_5_ at -3 T. In contrast to the large change of longitudinal resistivity *ρ_xx_* in high fields, here the Hall resistivity remains almost unchanged above the threshold electric field. Only a slight variation of relative resistivity (< 2%) is observed near the sliding transition possibly due to the small longitudinal resistivity component picked up by the Hall electrodes. We conclude that the low-field Hall effect of HfTe_5_ is not affected by the electric bias as expected for a density wave state. Figure S5c is the temperature dependence of nonlinear transport. The sliding transition behavior changes gradually from sharp to slow with the disappearance of two transition peaks upon the increase of temperature. Typically, such a peak-like behavior and sharp transitions appear in high-quality samples as reported earlier^9,10^. Hence, the strong temperature dependence of the sliding transition behavior may be related to the broadening of the Landau levels or the increase of electron kinetic energy. Figure S5d is the corresponding IV curves of Fig. S5c with clear nonlinear behavior. Figure S5e shows *|σ_xy_*| increases with *E*_b_ above the threshold electric field, suggesting the continuous activation of localized carriers.

**Figure S5. Magnetic field and temperature dependences of nonlinear transport.** (**a**) A comparison of the relative resistivity *ρ_xx_*/*ρ_xx_*_0_ (left panel) and quantum oscillations (right panel) with *ρ_xx_*_0_ being the differential resistivity value near zero *E*_b_. (**b**) The relative resistivity *ρ*/*ρ*_0_ at -3 T. (**c**) *ρ_xy_* as a function of the bias electric field at different temperatures at -20 T. (**d**) The *V*_xy_-*I* curves at different temperatures at 20 T, corresponding to the differential resistivity data in **c**. The brown dashed line is a linear guideline for comparison. (**e**) *|σ_xy_*| as a function of the biased electric field at -30 T and 1.5 K.

1. **Activation behavior of Hall conductivity in high fields**

The insulating phase of magnetic freeze-out at high magnetic fields comes from the carrier localization by impurities. As the temperature increases, the localized carriers become mobile again, resulting in an activation behavior on the carrier density^11^. The widely used Hall effect formula $\rho_{xy}=-\frac{B}{ne}$ does not include the magnetic field dependence of longitudinal resistivity. In high magnetic field regime ($\mu B\gg1$), the Hall conductivity formula $\sigma_{xy}=\frac{ne\mu^{2}B}{1+\mu^{2}B^{2}}\approx\frac{ne}{B}$ gives a better description of the transport properties. In Figs. S6 a-b, we present the conductivity tensor obtained through converting of the resistivity tensor shown in Fig. 2. In low temperature and high magnetic field conditions, $\sigma_{xx}\gg\sigma_{xy}$ and $\sigma_{xx}$ gradually saturates as *B* grows. Then the Hall resistivity is given by $\rho_{xy}=-\frac{\sigma_{xy}}{\sigma_{xx}^{2}+\sigma_{xy}^{2}}\approx-\frac{\sigma_{xy}}{\sigma_{xx}^{2}}$. In this case, both $|\rho_{xy}|$ and $|\sigma_{xy}|$ will decrease when reducing the carrier density *n*. The inset of Fig. R4b shows $\sigma_{xy}$ plotted with 1/*B*. In high field regime (small 1/*B*), $\sigma_{xy}$ deviates from the linear dependence with 1/*B* at low temperatures and drops toward zero, suggesting the decrease of mobile carriers.

Figure S6c shows the enlarged view of MR curves for 1.5~6 K with a crossing point at 11.8 T. The field-induced metal-insulator transition can be quantitatively analyzed using a scaling relation of $\rho_{xx}$ with *T* and *B* in the vicinity of the crossing point. Figure S6d presents the normalized resistivity $\rho_{xx}(B)/\rho_{xx}(B_{c})$ as a function of scaling parameter $|B-B_{c}|T^{-1/\zeta}$. The MR curves near the metal-insulator transition point can be well fitted by the scaling relation and yields a scaling parameter of $\zeta^{-1}=0.069$.

In Fig. S7, we show the comparison of the temperature dependences of *σ_xx_* and *σ_xy_* in three magnetic fields (30 T, 20 T, and 10 T). At 10 T (see Fig. S7c) where the magnetic freeze-out insulating phase has not yet started, both longitudinal and Hall components of the conductivity tensor show a similar systematic increase and finally get saturated with the temperature decreasing from 50 K to 1.5 K. But when entering the insulating phase (20 T and 30 T, see Figs. S7 b and a, respectively), *σ_xx_* still shows the increase-then-saturate behavior while *σ_xy_* dramatically decreases below 20 K due to the suppression of the carrier density. Note that according to the Hall conductivity, the carrier density decreases to nearly one-tenth from 15 K to 1.5 K, while *σ_xx_* increases by over 20%. It suggests that there may be other conducting channels that contribute to *σ_xx_* but not to *σ_xy_*. Previous angle-resolved photoemission spectroscopy (ARPES) measurements on similar HfTe_5_ samples (*T*_P_=65 K) showed no sign of other bands near Fermi level except for a Dirac-type band around the Γ point below 50 K^12^. One possible scenario for such conducting channels is the one-dimensional edge modes that do not contribute to Hall effect as proposed recently in KHgSb^13^. The overall behavior of both *σ_xx_* and *σ_xy_* in HfTe_5_ in high magnetic fields is quantitatively similar to that of KHgSb. For HfTe_5_, its single layer form has been predicted to be a large-gap quantum spin Hall insulator^14^. Here in magnetic fields, the inter-layer coupling of HfTe_5_ is suppressed by the formation of a spin density wave. It is possible that in high fields, when the bulk carriers are pinned to the impurities, the quantum spin Hall modes are responsible for the residue value of *σ_xx_* in the low-temperature limit. Further experiments at higher magnetic fields and lower temperatures will be helpful to check this assumption.

**Figure S6. The magnetic field dependence of longitudinal and Hall conductivity in HfTe_5_.** (**a-b**) *σ_xx_* (**a**) and *σ_xy_* (**b**) as a function of magnetic field at different temperatures. The magnetic field is applied along the *b* direction. The inset of **b** is *σ_xy_* as a function of 1/*B* at different temperatures. (**c-d**) The enlarged views of MR curves near the crossing points (**c**) and the scaling analysis (**d**).

**Figure S7. Temperature dependence of conductivity tensors.** (**a-c**) The temperature dependence of conductivity tensor components *σ_xx_* and *σ_xy_* at different magnetic fields (**a** for 30 T, **b** for 20 T and **c** for 10 T).

1. **Theoretical analysis of the field-induced density wave and magnetic freeze-out states**
2. **Density wave instability of Landau level**

As the low-temperature phase of HfTe_5_ is close to a Dirac semimetal, we consider that the low-energy physics is described by a massive Dirac Hamiltonian, which reads ($\hbar$ = 1)

$$H\left( \mathbf{k} \right)=v_{a}k_{a}\tau_{x}\bigotimes s_{x}+v_{c}k_{c}\tau_{x}\bigotimes s_{y}+v_{b}k_{b}\tau_{x}\bigotimes s_{z}+m\tau_{z}, (1)$$

where $s_{i}$ and $\tau_{i}$ with $i=\left\{ x, y, z \right\}$ are Pauli matrices acting on spin space and orbital space, respectively. $k_{\alpha}$ with $\alpha=\left\{ a, b, c \right\}$ correspond to the momenta along the three principal axes, and *v* represents the corresponding Fermi velocity, which, without losing generality, is assumed to be positive for the brevity of notation. Imposing a magnetic field applied along the *b* direction, the energy spectra of the massive Dirac Hamiltonian are discretized into Landau bands, which read

$$E_{n,\pm}\left( k_{b} \right)=\pm\sqrt{m^{2}+v_{b}^{2}k_{b}^{2}+2v_{a}v_{c}neB}, (2)$$

where *B* denotes the magnetic field, and $n=\{0, 1, 2, \ldots\}$ labels the Landau bands. It is worth stressing that all Landau bands are doubly degenerated except for the zeroth ones which are spin-polarized, as illustrated in Fig. S2. Furthermore, because of the existence of spin-orbit coupling, the orbital polarization of the zeroth Landau levels depends on the momentum. To see this, we write down the eigenvectors for the zeroth Landau levels explicitly, which are

$|u_{0,+}(k_{b})\rangle=\left(\begin{matrix} \cos\frac{\theta}{2} \\ 0 \\ \sin\frac{\theta}{2} \\ 0 \end{matrix}\right)$ , $|u_{0,-}(k_{b})\rangle=\left(\begin{matrix} \sin\frac{\theta}{2} \\ 0 \\ -\cos\frac{\theta}{2} \\ 0 \end{matrix}\right)$, $\theta=\arctan\frac{v_{b}k_{b}}{m}$ . (3)

Accordingly, one can find

$$\langle\text{u}_{0,+}(k_{b})|\tau_{0}\otimes s_{z}|\text{u}_{0,+}(k_{b})\rangle=1,\langle\text{u}_{0,+}(k_{b})|\tau_{0}\otimes s_{y}|\text{u}_{0,+}(k_{b})\rangle=0,$$

$$\langle\text{u}_{0,+}(k_{b})|\tau_{0}\otimes s_{x}|\text{u}_{0,+}(k_{b})\rangle=0,\langle\text{u}_{0,+}(k_{b})|\tau_{z}\otimes s_{0}|\text{u}_{0,+}(k_{b})\rangle=\frac{m}{\sqrt{m^{2}+(v_{b}k_{b})^{2}}},$$

$$\langle\text{u}_{0,+}(k_{b})|\tau_{x}\otimes s_{0}|\text{u}_{0,+}(k_{b})\rangle=\frac{v_{b}k_{b}}{\sqrt{m^{2}+(v_{b}k_{b})^{2}}},\langle\text{u}_{0,+}(k_{b})|\tau_{y}\otimes s_{0}|\text{u}_{0,+}(k_{b})\rangle=0;$$

$$\langle\text{u}_{0,-}(k_{b})|\tau_{0}\otimes s_{z}|\text{u}_{0,-}(k_{b})\rangle=1,\langle\text{u}_{0,-}(k_{b})|\tau_{0}\otimes s_{y}|\text{u}_{0,-}(k_{b})\rangle=0,$$

$$\langle\text{u}_{0,-}(k_{b})|\tau_{0}\otimes s_{x}|\text{u}_{0,-}(k_{b})\rangle=0,\langle\text{u}_{0,-}(k_{b})|\tau_{z}\otimes s_{0}|\text{u}_{0,-}(k_{b})\rangle=-\frac{m}{\sqrt{m^{2}+(v_{b}k_{b})^{2}}},$$

$\langle\text{u}_{0,-}(k_{b})|\tau_{x}\otimes s_{0}|\text{u}_{0,-}(k_{b})\rangle=-\frac{v_{b}k_{b}}{\sqrt{m^{2}+(v_{b}k_{b})^{2}}},\langle\text{u}_{0,-}(k_{b})|\tau_{y}\otimes s_{0}|\text{u}_{0,-}(k_{b})\rangle=0.$ (4)

According to the above results, one can find that the two zeroth Landau levels are spin-polarized but the orbital polarization depends on the momentum. In the limit of zero Dirac mass, i.e., $m=0$, the two zeroth Landau levels correspond to two chiral Landau levels with opposite chirality and orbital polarization. The degeneracy of higher Landau levels will be lifted with further consideration of the Zeeman splitting effect, but here, as the region we are interested in corresponds to the low-field regime, we first neglect the effect of Zeeman splitting for simplicity.

In the following, we focus on the quantum-limit regime in which the electrons only occupy the spin-polarized zeroth Landau levels. Because the zeroth Landau levels are spin-polarized, the relevant Coulomb interaction and electron-phonon interaction respectively take the form

$$H_{\text{Coulomb}}=\sum_{k,k',q,\alpha,\beta} V_{\alpha\beta}(k,k'-q)c_{\alpha,k+q,\uparrow}^{+}c_{\beta,k'-q,\uparrow}^{+}c_{\beta,k',\uparrow}c_{\alpha,k,\uparrow},$$

$H_{\text{e}-\text{ph}}=\sum_{q} \omega(q)b_{q}^{+}b_{q}+\sum_{k,q,\alpha,\beta} M_{\alpha\beta}(q)c_{\alpha,k,\uparrow}^{+}c_{\beta,k+q,\uparrow}(b_{q}+b_{-q}^{+}).$ (5)

Above $\left\{\alpha\text{，}\beta\right\}$ represent the two orbital degrees of freedom. Projecting onto the upper zeroth Landau level, the interactions become

$$H_{\text{Coulomb}}=\sum_{k,k',q} \overline{V}(k,k'-q)c_{+,k+q,\uparrow}^{+}c_{+,k'-q,\uparrow}^{+}c_{+,k',\uparrow}c_{+,k,\uparrow}+...,$$

$H_{\text{e}-\text{ph}}=\sum_{q} \omega(q)b_{q}^{+}b_{q}+\sum_{k,q} \overline{M}(q)c_{+,k,\uparrow}^{+}c_{+,k+q,\uparrow}(b_{q}+b_{-q}^{+})+...,$ (6)

where the subscript “+” labels the upper zeroth Landau level, and “…” stands for interactions involving electrons from both zeroth Landau levels. The two interaction coeffcients are given by

$$\begin{matrix} \overline{V}(k,k'-q)=V_{11}(k,k'-q)\cos\frac{\theta_{k+q}}{2}\cos\frac{\theta_{k'-q}}{2}\cos\frac{\theta_{k'}}{2}\cos\frac{\theta_{k}}{2}+V_{22}(k,k'-q)\sin\frac{\theta_{k+q}}{2}\sin\frac{\theta_{k'-q}}{2}\sin\frac{\theta_{k'}}{2}\sin\frac{\theta_{k}}{2} \\ +V_{12}(k,k'-q)\cos\frac{\theta_{k+q}}{2}\sin\frac{\theta_{k'-q}}{2}\sin\frac{\theta_{k'}}{2}\cos\frac{\theta_{k}}{2}+V_{21}(k,k'-q)\sin\frac{\theta_{k+q}}{2}\cos\frac{\theta_{k'-q}}{2}\cos\frac{\theta_{k'}}{2}\sin\frac{\theta_{k}}{2}, \end{matrix}$$

$\begin{matrix} \overline{M}(q)=M_{11}(q)\cos\frac{\theta_{k}}{2}\cos\frac{\theta_{k+q}}{2}+M_{22}(q)\sin\frac{\theta_{k}}{2}\sin\frac{\theta_{k+q}}{2} \\ +M_{12}(q)\cos\frac{\theta_{k}}{2}\sin\frac{\theta_{k+q}}{2}+M_{21}(q)\sin\frac{\theta_{k}}{2}\cos\frac{\theta_{k+q}}{2}. \end{matrix}$ (7)

Let us first give a discussion about the coefficient of electron-phonon interaction. Assume that the Fermi level crosses the upper zeroth Landau level at momentum $\pm k_{F}$ and the density wave instability originates from the nesting. As the nesting vector $q=2k_{F}$ connects the left Fermi point at $-k_{F}$ to the right Fermi point $k_{F}$, and in the limit of zero Dirac mass, $\theta_{k}\to\pm\pi/2$, depending on the sign of $k$, it is easy to find

$\overline{M}\left(2k_{F}\right)\sim\frac{1}{2}[M_{11}\left(2k_{F}\right)-M_{22}\left(2k_{F}\right)+M_{12}\left(2k_{F}\right)-M_{22}\left(2k_{F}\right)]$. (8)

Therefore, if the coefficients of electron-phonon interaction are symmetric about the orbital indices, i.e., $M_{11}(q)=M_{22}(q),M_{12}(q)=M_{21}(q)$, then $\overline{M}\left(2k_{F}\right)\sim0$. This result suggests that because the zeroth Landau levels have momentum-dependent orbital polarization, the electron-phonon interaction is not favored to drive the density wave instability if the Dirac mass is close to zero and the coefficients of electron-phonon interaction are symmetric about the orbital indices. Similar analysis can be performed for the Columb interaction, one can find

$\overline{V}\left(2k_{F}\right)\equiv\overline{V}\left(-k_{F},-k_{F}\right)\sim\frac{1}{4}[V_{11}\left(-k_{F}\right)+V_{22}\left(-k_{F}\right)-V_{12}\left(-k_{F}\right)-V_{21}\left(-k_{F}\right)]$. (9)

Due to the Pauli exclusion principle and the difference in the extension of wave functions for electrons with different orbitals, the intra-orbital coefficients are in general distinct to the inter-orbit coefficients, leading to a nonzero $\overline{V}(2k_{F})$. Comparing the two interactions at the nesting vector, it seems that the Coulomb interaction, in general, is favored to drive the density-wave instability if the Dirac mass is close to zero.

No matter whether the density wave instability is driven by Coulomb interaction or electron-phonon interaction, the picture for the quantized Hall plateaus is qualitatively the same. In the following, let us assume that the density wave has formed and just focus on the quantized Hall conductivity/resistivity. For Landau levels, it is known that the low-energy Hamiltonian close to the Fermi points are given by

$H=\sum_{k} v_{n,\beta}\left( k-k_{F,n,\beta} \right)c_{k,n,\beta}^{\dagger}c_{k,n,\beta},$(10)

where $\beta=-,+$, corresponding to the left-moving and right-moving modes, respectively; $k_{F,n,\beta}$ refers to the Fermi momentum of the *n*-th Landau levels, which takes the form

$k_{F,n,\beta}=\frac{\beta}{v_{b}}\sqrt{E_{F}^{2}-m^{2}-2v_{a}v_{c}neB}.$(11)

Correspondingly,

$v_{n,\beta}=\frac{v_{b}^{2}k_{F,n,\beta}}{E_{F}}.$(12)

The density of the carriers is given by

$\rho=\frac{eB}{2\pi}\sum_{n} \int\frac{dk_{b}}{2\pi}\Theta\left( E_{F}-E_{n,+}\left( k_{b} \right) \right)=\frac{eB}{2\pi^{2}}\left[ k_{F,0}+2\sum_{n=1}^{n_{\max}} k_{F,n} \right],$ (13)

where $k_{F,n}={(E_{F}^{2}-m^{2}-2v_{a}v_{c}neB)}^{1/2}/v_{b}$, and $n_{\max}$ corresponds to the integer fulfilling $(E_{F}^{2}-m^{2})\geq2v_{a}v_{c}n_{\max}eB$ but $(E_{F}^{2}-m^{2})\leq2v_{a}v_{c}(n_{\max}+1)eB$. The factor of 2 in the bracket counts the double degeneracy of Landau bands for $n\geq1$. Assuming that the density of carriers does not change with the magnetic field, it is apparent that the Fermi level will vary with the magnetic field. If we define

$Q_{F}=2\left[ k_{F,0}+2\sum_{n=1}^{n_{\max}} k_{F,n} \right]=\frac{4\pi^{2}\rho}{eB},$ (14)

it is apparent that this quantity decreases continuously with the increase of the magnetic field.

In a seminal paper, Halperin pointed out that when the Fermi level is located at the density-wave-induced gap, the Hall conductivity in the plane perpendicular to the density wave direction (here along the magnetic field direction) is quantized and given by^15^

$\sigma_{ac}=\frac{e^{2}}{h}\frac{G_{d}}{2\pi},$ (15)

where $G_{d}$ represents the wave vector of the density wave. Thus, if assuming that all Landau levels undergo density wave instability simultaneously and the Fermi level is located at all density-wave induced gaps, the Hall conductivity is given by

$\sigma_{ac}=\frac{e^{2}}{h}\frac{Q_{F}}{2\pi},$(16)

while the corresponding Hall resistivity is $\rho_{ac}=\sigma_{ac}^{-1}$. It is sensible to assume that once the density wave instability takes place, *Q_F_* is fixed for an interval of the magnetic field, thus forming a Hall plateau.

Within the framework of Landau-level nesting, the density wave instability of Landau levels will only result in a one-dimensional density wave whose wave vector is along the magnetic field direction. Taking into account the impurity pinning effect, this will result in nonlinear transports only along the magnetic field direction. However, the in-plane nonlinear transports observed in our experiment suggest that there is a field-induced density wave order along the direction *a*. The remarkable inconsistency between the experimental results and the theoretical predictions based on the Landau-level nesting scenario suggests that another scenario for the density wave is needed. In the following, we will show that the quantized nesting model can well account for the key experimental results in the quantum Hall regime.

1. **Quantized nesting model**

It is known that a three-dimensional density wave order can be induced by magnetic fields in Bechgaard salts^16^. The Bechgaard salts are quasi-one-dimensional metals with open Fermi surface, when a magnetic field is applied in the direction with the weakest dispersion, the materials will undergo a series of spin density wave transitions and display a series of Hall plateaus^17–19^. As HfTe_5_ is also very anisotropic in band structure and the magnetic field is also applied along the direction with the weakest dispersion, it is reasonable to expect that similar field-induced spin density wave may also take place in this material.

Now we apply the quantized nesting model to HfTe_5_. As the nonlinear transport behaviors observed in the experiment suggest that the nesting momentum is a three-dimensional vector, in the following we will make use of the tight-binding Hamiltonian instead of the continuum one in Eq.(1). Concretely, the tight-binding counterpart of Eq.(1) reads

$H_{0}\left( \vec{k} \right)=\left[ m_{0}-t_{a}\cos\left( k_{a}a \right)-t_{b}\cos\left( k_{b}b \right)-t_{c}\cos\left( k_{c}c \right) \right]\tau_{z}\bigotimes s_{0}$

$+\lambda_{a}\sin\left( k_{a}a \right)\tau_{x}\bigotimes s_{x}+\lambda_{b}\sin\left( k_{b}b \right)\tau_{x}\bigotimes s_{y}+\lambda_{c}\sin\left( k_{c}c \right)\tau_{x}\bigotimes s_{z},$ (17)

where the basis is $\psi_{k}={(c_{1,k},c_{2,k},c_{3,k},c_{4,k})}^{T}$, and $\lambda_{i=a,b,c}$ characterize the strength of spin-orbit coupling. When $\lambda_{i}\ll t_{i},$ the spin-orbit coupling has a much more important impact on the band topology than the band dispersion. By a unitary transformation, $\tilde{\psi}_{k}={(\tilde{c}_{1,k},\tilde{c}_{2,k},\tilde{c}_{3,k},\tilde{c}_{4,k})}^{T}=U_{k}\psi_{k}$, the Hamiltonian can be diagonalized in the form

$H_{0}=\sum_{k} \tilde{\psi}_{k}^{\dagger}[\sqrt{M^{2}\left( k \right)+d^{2}(k)}\tilde{\tau}_{0}\bigotimes\tilde{s}_{z}]\tilde{\psi}_{k}$, (18)

where the Pauli matrices $\tilde{\tau}_{i}$ and $\tilde{s}_{i}$ act on the two degenerate conduction and valence bands, respectively, and $M\left( k \right)=m_{0}-t_{a}\cos\left( k_{a}a \right)-t_{b}\cos\left( k_{b}b \right)-t_{c}\cos\left( k_{c}c \right)$ and $d\left( k \right)=\sqrt{{[\lambda_{a}\sin\left( k_{a}a \right)]}^{2}+{[\lambda_{b}\sin\left( k_{b}b \right)]}^{2}+{[\lambda_{c}\sin\left( k_{c}c \right)]}^{2}}$. In such a band basis, the band-topology information is encoded in the transformation matrix $U_{k}$.

Let us now further take into account the interaction. Similar to the superconducting instability, the density-wave instability is an instability of the Fermi surface, therefore, within the weak-coupling regime, one can just focus on the two degenerate bands crossing the Fermi level. Without loss of generality, here we consider the Fermi level crosses the two conduction bands. To get the relevant full Hamiltonian, we need to further project the interaction onto the band basis. As currently, the driving force of the density-wave instability is still unclear, here just for illustration, we assume that the driving force is the electron-electron interaction. On the original basis, the general form of the interaction reads

$H_{coulomb}=\sum_{k,k^{'},q;\alpha,\beta} V_{\alpha\beta}(k,k^{'}-q)c_{\alpha,k+q}^{\dagger}c_{\beta,k^{'}-q}^{\dagger}c_{\beta,k'}c_{\alpha,k}$ (19)

where $\alpha$ and $\beta$ run over 1,2,3,4. By using the unitary matrix $U_{k}$, the projected interaction responsible for the density wave instability will take the form

$H_{coulomb}=\sum_{k,k^{'},q;\tau,\tau'} \tilde{V}_{\tau\tau'}(k,k^{'}-q)\tilde{c}_{\tau,k+q}^{\dagger}\tilde{c}_{\tau',k^{'}-q}^{\dagger}\tilde{c}_{\tau',k'}\tilde{c}_{\tau,k}$, (20)

where $\tau$ and $\tau'$ only run over 1,2, the band index of the two conduction bands, and

$\tilde{V}_{\tau\tau^{'}}\left( k,k^{'}-q \right)=\sum_{\alpha,\beta} V_{\alpha\beta}(k,k^{'}-q)\left( U_{k+q} \right)_{\tau\alpha}\left( U_{k^{'}-q} \right)_{\tau'\beta}\left( U_{k'}^{\dagger} \right)_{\tau'\beta}\left( U_{k}^{\dagger} \right)_{\tau\alpha}$. (21)

One can see that, in the band basis, the band topology encoded in the wave functions is now transferred to the interaction. According to the above analysis, the Hamiltonian responsible for the density-wave instability is given by

$H=\sum_{k,\tau} [\sqrt{M^{2}\left( k \right)+d^{2}\left( k \right)}-\mu]\tilde{c}_{\tau,k}^{\dagger}\tilde{c}_{\tau,k}$

+$\sum_{k,k^{'},q;\tau,\tau'} \tilde{V}_{\tau\tau'}(k,k^{'}-q)\tilde{c}_{\tau,k+q}^{\dagger}\tilde{c}_{\tau',k^{'}-q}^{\dagger}\tilde{c}_{\tau',k'}\tilde{c}_{\tau,k}$.(22)

As we mentioned above, when the energy scale associated with the spin-orbit coupling is much smaller than that of other hoppings,the spin-orbit coupling has a much more important impact on the band topology than the band dispersion. As in the band basis, the information of the band topology is now transferred to the interaction, we can ignore the weak modifications from the spin-orbit coupling to the band dispersion, accordingly, the Hamiltonian can be reduced as

$H\approx\sum_{k,\tau} [M(k)-\mu]\tilde{c}_{\tau,k}^{\dagger}\tilde{c}_{\tau,k}$+$\sum_{k,k^{'},q;\tau,\tau'} \tilde{V}_{\tau\tau'}(k,k^{'}-q)\tilde{c}_{\tau,k+q}^{\dagger}\tilde{c}_{\tau',k^{'}-q}^{\dagger}\tilde{c}_{\tau',k'}\tilde{c}_{\tau,k}$. (23)

By linearizing the direction with the strongest dispersion, *i.e.*,

$M\left( k \right)-\mu\approx\pm v_{F}\left( k_{a}\mp k_{F} \right)-t_{b}\cos\left( k_{b}b \right)-t_{c}\cos\left( k_{c}c \right)$, (24)

the Hamiltonian goes to the form of the standard quantized nesting model^20^. What is essentially different here is that the interaction term now contains the information of the band topology. A potential consequence of the nontrivial band topology is that the order parameter of the resulting density wave may develop nontrivial phase dependence.

As we have shown that the interacting Hamiltonian can be mapped to the quantized nesting model, it is reasonable to follow the quantized nesting model to explain the observed experimental results. According to Eq. (24), the dispersions have perfect nesting, with the nesting vector $\overrightarrow{Q}=(2k_{F},\pi/b,\pi/c)$, therefore, a density wave instability which opens a full gap on the Fermi surface would happen after taking into account the Coulomb interaction. However, it is worth noting that the above dispersions with perfect nesting are a rough approximation of the real dispersions. As the real dispersions do not have perfect nesting, the density wave in fact can only gap a part of the Fermi surfaces, leaving closed electron and hole pockets behind^20^. Without the magnetic field, since after the occurrence of density wave instability, the system remains metallic, the gain in elastic energy will generally win the reduction of energy in electronic energy, indicating that the density wave is not stable in the absence of magnetic field^20^. However, in the presence of magnetic fields, the closed electron and hole pockets will be discretized into Landau levels. When the Fermi level is simultaneously located within the spin-density-wave gap and the gap between two Landau levels, the spin density wave can be stabilized^20^, and importantly, with the increase of magnetic field, the spin density wave can adjust the density wave vector to $\overrightarrow{Q}=(2k_{F}+nG,\pi/b,\pi/c)$ ($G=eHc/v_{c}$with $H$ the magnetic field strength, $v_{c}$ the velocity of light and c the lattice constant in the direction $\overrightarrow{c}$)^21^, so that the Fermi level remains in the gap, leading to the emergence of a Hall plateau^20^. When the Fermi level crosses the Landau levels with the increase of magnetic field, the spin density wave will become unstable, consequently leading to a cascade of phase transitions between spin density waves with distinct wave vectors. Accompanying these transitions, the Hall resistivity of the Hall plateaus changes, with their ratio following $...:\frac{1}{5}:\frac{1}{4}:\frac{1}{3}:\frac{1}{2}:1$ if the electron and hole pockets consist of conventional quasiparticles, and $...:\frac{1}{9}:\frac{1}{7}:\frac{1}{5}:\frac{1}{3}:1$ if they consist of Dirac quasiparticles. It is because the corresponding zeroth Landau levels of Dirac quasiparticles are spin-polarized, while other higher Landau levels are doubly degenerate. Remarkably, in our experiment, three Hall plateaus are observed (see Figure1b of the main text), and the ratio of Hall resistivity $\rho_{3}:\rho_{2}:\rho_{1}=\frac{1}{5.2}:\frac{1}{3.1}:1\approx\frac{1}{5}:\frac{1}{3}:1$, consistent with the massive Dirac band nature in HfTe_5_.

Compared to the Landau-level nesting scenario, one can find that according to the quantized nesting model, the density wave vector is a three-dimensional vector. Accordingly, the spin density will be modulated in all three directions. The presence of impurity will modulate the phase of a spin density wave in all three directions, resulting in the pinning of spin density wave in all three directions. This is consistent with our experimental observation of the presence of prominent nonlinear transports in the directions perpendicular to the magnetic field.

Here we would like to give a discussion about the difference in nonlinear transports between Bechgaard salts and HfTe_5_. Compared to the Bechgaard salts, HfTe_5_ hosts a closed Fermi surface instead of being open. The closed Fermi surface suggests that the nesting of Fermi surface in HfTe_5_ should be weaker than that in Bechgaard salts, but this should mainly affect the in-plane density wave, as only in the *a-c* plane the anisotropy of Fermi surface is relatively weak in HfTe_5_. We find that it is consistent with the experimental results, which show that in the presence of an in-plane biased electric field, the change of the in-plane resistivity in HfTe_5_ is small, indicating that the electrons are locked by the in-plane density wave are a small amount.

Before ending this subsection, we would like to give a further discussion of the nonvanishing longitudinal magnetoresistance in the whole quantum Hall regime. It is known that the presence of Hall plateaus with concomitant dips in longitudinal magnetoresistance is the evidence of the quantum Hall effect. In 2D systems, the dips commonly reach zero when the temperature is sufficiently low. However, in 3D systems, the dips commonly do not reach zero even at very low temperatures, especially for the ones corresponding to the plateaus in the low-field regime. Through the study of the quantum Hall effect in two dimensions, it is known that disorder-induced localization of electrons is necessary to form the exact Hall plateau. Meanwhile, there exists a constraint on the number of disorders that the broadening of the Landau level induced by disorders should be smaller than the energy gap between adjacent Landau levels. In two dimensions, this is easy to satisfy since the scaling theory of localization^22^ indicates that the electrons are always inclined to localize in the presence of disorder, irrespective of the strength of disorders. In contrast, in 3D systems, the localization of electrons requires the strength of disorders to exceed a material-dependent critical value. In addition, for the field-induced spin density wave, impurities can induce extended states even when the Fermi level is in the gap^23^. As a result of these facts, there in principle exist a small number of delocalized electrons even in the quantum Hall regime. Since the delocalized electrons in bulk are dissipative in transport, the longitudinal magnetoresistance tends to deviate from zero.

1. **Field-induced magnetic freeze-out**

In the absence of magnetic field, Mott found that in a doped semiconductor, as the density of electrons drops, bound states become possible when

$$\rho^{1/3}a_{B}=0.25, (25)$$

where $\rho$ is the density of electrons, and $a_{B}$ is the Bohr radius for a donor (an impurity site with a positive charge)^24^. As the density of electrons further decreases, the electrons will become trapped at the impurity sites, consequently the number of mobile carriers decreases, and a metal-insulator transition takes place.

When the density of electrons is fixed, such a metal-insulator transition is also possible in the presence of magnetic field. In a sufficiently high magnetic field, the impurity levels become deep enough to cause the localization of carriers at the impurity sites, resulting in the so-called freeze-out of mobile carriers. In this case, similarly, the transition takes place when

$${(\rho l_{m}^{2}a_{b})}^{1/3}\sim0.3, (26)$$

where $l_{m}=\sqrt{\frac{\hbar}{eB}}$ is the magnetic length, and $a_{b}=\frac{4\pi\hbar^{2}\varepsilon_{0}\varepsilon}{m_{b}^{*}e^{2}}$ denotes the Bohr radius in the *b* direction with $m_{b}^{*}$ the corresponding effective mass.

In our experiment, $\rho=2.7\times{10}^{17} \mathrm{cm}^{-3}$. The effective mass along the b direction can be calculated as $m_{b}^{*}=\frac{m_{a-b}^{*}m_{b-c}^{*}}{m_{a-c}^{*}}=1.27m_{e}$. Then it is readily obtained that

$$B_{c}\approx8.2 T, (27)$$

which is close to the experimental result of ~11.8 T.

Now we give a discussion of the binding energy. As the donor has an inner structure that the electron cannot penetrate due to the Pauli exclusion principle, it is sensible to assume that the Coulomb potential takes a hard-core form,

$$V\left( r \right)=\left\{ \begin{matrix} \frac{A}{r}, r>a_{0}, \\ 0, r<a_{0}, \end{matrix} \right. (28)$$

where $A=-e^{2}/(4\pi\varepsilon_{0}\varepsilon)$, and $a_{0}$ is a characteristic length of the hard-core structure. According to this form, when the magnetic length $l_{m}\gg a_{0}$, and $l_{m}\ll a_{\mathrm{plane}}$ ($a_{\mathrm{plane}}=\frac{4\pi\hbar^{2}\varepsilon_{0}\varepsilon}{\sqrt{m_{a}m_{c}}e^{2}}$), the binding energy can be approximated as

$$\varepsilon_{b}\sim\left| V\left( l_{m} \right) \right|\propto\sqrt{B}. (29)$$

As the magnetic field increases, $l_{m}$ gets close to $a_{0}$ gradually, then the radius of the electrons will no longer shrink due to the Pauli exclusion principle, instead, it will saturate. As a result, the binding energy also saturates, qualitatively agreeing with the experimental observation.

The corresponding impurity band responsible for the magnetic freeze-out effect can be roughly estimated using the magnetic-field-dependent Fermi energy. In four samples we measured in high field experiments (see Fig. 2 and Fig. S3 for sample H2 and H3), the critical field of Hall resistivity peak ranges from 9.5~12 T. Taking B=11 T (the value of sample H2) as the onset field of the magnetic freeze-out effect, the energy of impurity band can be estimated as $E_{I}=E_{F}\left( B \right)=\sqrt{\left( \frac{\Delta}{2} \right)^{2}+\left( v_{F}\hbar k_{F} \right)^{2}}-\frac{g\mu_{B}B}{2}\approx4.1 \mathrm{meV}$.

To further exclude the Wigner crystal mechanism, we provide an estimation of the upper bound for the critical temperature by following the phase diagram provided in the reference^25^. The electron density of HfTe_5_ (~2.7×10^17^cm^-3^) sets an upper bound of ~9 K for the critical transition temperature of the Wigner crystal phase^25^. Meanwhile, it is worth noting that the phase diagram in this reference is based on electrons whose masses are not renormalized. In HfTe_5_, the effective mass is very small as it is close to the phase boundary of a strong topological insulator and a weak topological insulator. The smallness of effective mass will enhance the average kinetic energy, consequently reducing the critical temperature. Hence, the critical temperature for the realization of Wigner crystal phase in HfTe_5_ should be much less than 9 K.

Another way to estimate the critical temperature is to follow the experiment which first observed the formation of the Wigner crystal phase on the surface of liquid Helium^26^. In this reference, the authors treated the electrons on the two-dimensional surface as classical particles. Accordingly, the average kinetic energy per particle is *k*_B_*T*, where *k*_B_ is the Boltzmann constant and *T* is the temperature. The average Coulomb potential energy per particle is $\frac{e^{2}}{4\pi\varepsilon_{0}r_{e}}$, where $r_{e}=\frac{1}{\sqrt{\pi n_{2d}}}$ is the average radius of electrons and $n_{2d}$ is the electron density. The ratio of Coulomb potential energy to kinetic energy per particle, defined as $\Gamma=\frac{{{n_{2d}}^{\frac{1}{2}}e}^{2}}{4\pi^{\frac{1}{2}}\varepsilon_{0}k_{B}T}$, determines the extent of correlation effects. According to the experimental finding^26^, the Wigner crystal is realized at $\Gamma=173$. For HfTe_5_ concerned here, as the material is weakly coupled along the *b*-axis, we can approximately treat the system as a stacking of two-dimensional layers. For each layer, the density is $n_{2d}=n_{3d}l_{b}\approx4\times{10}^{10} \mathrm{cm}^{-2}$ with $l_{b}$ the interlayer distance. Then the critical temperature for the Wigner crystal can be estimated as $T=\frac{{{n_{2d}}^{\frac{1}{2}}e}^{2}}{4\pi^{\frac{1}{2}}\varepsilon_{0}\times137k_{B}}\approx4 K$. Since the effective mass for HfTe_5_ should be smaller than that on the surface of liquid Helium^27^ and the correlation effect is expected to decrease after taking the third dimension into account. Then the real critical temperature for the realization of the Wigner crystal phase in HfTe_5_ should be considerably smaller than this estimated temperature.

Both methods yield a critical temperature much less than 10 K. In experiments, the suppression of Hall conductivity at high fields starts around 40 K. Therefore, the high-field insulating phase should not come from the formation of Wigner crystals.

1. **Possible edge states in the presence of magnetic field**

As an example, we consider that the system is in the strong topological insulator phase. So at each face of a cubic sample, there is a single Dirac cone. Let us focus on the *c*-direction surfaces. According to the Hamiltonian in Eq. (1), the Dirac cones at these surfaces are described by

$$H_{\pm}\left( k_{a},k_{b} \right)=\mp v_{a}k_{a}s_{x}+v_{b}k_{b}s_{y}, (30)$$

where $s_{i}$ are Pauli matrices acting on the subspace spanned by two orthogonal vectors. For $H_{+}$, the two vectors are $\left. |\tau_{y}=1, s_{y}=1 \right\rangle$ and $\left. |\tau_{y}=-1, s_{y}=-1 \right\rangle$. For $H_{-}$, the two vectors are $\left. |\tau_{y}=1, s_{y}=-1 \right\rangle$ and $\left. |\tau_{y}=-1, s_{y}=1 \right\rangle$. Now consider applying the magnetic field along the *b* direction, in the original basis, the Zeeman field takes the form of $V_{z}s_{z}$. If we take the Zeeman field as a perturbation, it is readily found through the first-order perturbation theory that its contribution to the surface-state Hamiltonian is zero, in other words, the surface states on the *c*-direction surfaces will not be gapped out by the magnetic field along the *b* direction. Therefore, their energy spectra are given by

$$E\left( k_{a},k_{b} \right)=\pm\sqrt{\left( v_{a}^{2}k_{a}^{2}+v_{b}^{2}k_{b}^{2} \right)}, (31)$$

which stay gapless. As a result, they will contribute a series of channels for transport along the *a* direction if the Fermi energy crosses the surface-state spectra. As these boundary states do not conduct cyclotron motion in the *a-c* plane, they do not contribute to the Hall effect, they only contribute to the longitudinal transport.

1. **References**

1 Wang H, Li C-K, and Liu H *et al.* Chiral anomaly and ultrahigh mobility in crystalline HfTe_5_. *Phys Rev B* 2016; **93**: 165127.

2 Zhao L-X, Huang X-C, and Long Y-J *et al.* Anomalous Magneto-Transport Behavior in Transition Metal Pentatelluride HfTe_5_. *Chin Phys Lett* 2017; **34**: 037102.

3 Wang P, Ren Y, and Tang F *et al.* Approaching three-dimensional quantum Hall effect in bulk HfTe_5_. *Phys Rev B* 2020; **101**: 161201.

4 Galeski S, Zhao X, and Wawrzyńczak R *et al.* Unconventional Hall response in the quantum limit of HfTe_5_. *Nat Commun* 2020; **11**: 5926.

5 Bernevig BA, Hughes TL. *Topological insulators and topological superconductors*. Princeton University Press, Princeton, 2013.

6 Monçeau, P. P, Ong NP, and Portis AM *et al.* Electric Field Breakdown of Charge-Density-Wave-Induced Anomalies in NbSe_3_. *Phys Rev Lett* 1976; **37**: 602–606.

7 Grüner G. The dynamics of spin-density waves. *Rev Mod Phys* 1994; **66**: 1–24.

8 Grüner G, Zawadowski A, and Chaikin PM. Nonlinear Conductivity and Noise due to Charge-Density-Wave Depinning in NbSe_3_. *Phys Rev Lett* 1981; **46**: 511–515.

9 Thorne RE, Tucker JR, and Bardeen J. Experiment versus the classical model of deformable charge-density waves: Interference phenomena and mode locking. *Phys Rev Lett* 1987; **58**: 828–831.

10 Grüner G. The dynamics of charge-density waves. *Rev Mod Phys* 1988; **60**: 1129.

11 Dyakonov MI, Efros AL, and Mitchell DL. Magnetic Freeze-Out of Electrons in Extrinsic Semiconductors. *Phys Rev* 1969; **180**: 813–818.

12 Zhang Y, Wang C, and Liu G *et al.* Temperature-induced Lifshitz transition in topological insulator candidate HfTe_5_. *Sci Bull* 2017; **62**: 950–956.

13 Liang S, Kushwaha S, and Gao T *et al.* A gap-protected zero-Hall effect state in the quantum limit of the non-symmorphic metal KHgSb. *Nat Mater* 2019; **18**: 443–447.

14 Weng H, Dai X, and Fang Z. Transition-Metal Pentatelluride ZrTe_5_ and HfTe_5_: A Paradigm for Large-Gap Quantum Spin Hall Insulators. *Phys Rev X* 2014; **4**: 011002.

15 Halperin BI. Possible States for a Three-Dimensional Electron Gas in a Strong Magnetic Field. *Jpn J Appl Phys* 1987; **26**: 1913.

16 Gor’kov LP, Lebed’ AG. On the stability of the quasi-one-dimensional metallic phase in magnetic fields against the spin density wave formation. *J Phys Lett* 1984; **45**: 433–440.

17 Héritier, M., Montambaux, G., and Lederer, P. Stability of the spin density wave phases in (TMTSF)_2_ClO_4_ : quantized nesting effect. *J Phys Lett* 1984; **45**: 943–952.

18 Poilblanc D, Montambaux G, and Héritier M *et al.* Quantized Hall effect in the field-induced density-wave phases of low-dimensionality conductors. *Phys Rev Lett* 1987; **58**: 270–273.

19 Hannahs ST, Brooks JS, and Kang W *et al.* Quantum Hall effect in a bulk crystal. *Phys Rev Lett* 1989; **63**: 1988–1991.

20 Chaikin PM. Field Induced Spin Density Waves. *J Phys I* 1996; **6**: 1875–1898.

21 Yakovenko VM. Quantum Hall effect in quasi-one-dimensional conductors. *Phys Rev B* 1991; **43**: 11353.

22 Abrahams E, Anderson PW, and Licciardello DC *et al.* Scaling Theory of Localization: Absence of Quantum Diffusion in Two Dimensions. *Phys Rev Lett* 1979; **42**: 673–676.

23 Azbel MY, Bak P, and Chaikin P. Open orbits and generalized quantum Hall effect. *Phys Rev Lett* 1987; **59**: 926.

24 Mott N. Metals, nonmetals and metal-nonmetal transitions: some recollections. *Rep Prog Phys* 1984; **47**: 909–923.

25 Ceperley D. Return of the itinerant electron. *Nature* 1999; **397**: 386–387.

26 Grimes CC, Adams G. Evidence for a Liquid-to-Crystal Phase Transition in a Classical, Two-Dimensional Sheet of Electrons. *Phys Rev Lett* 1979; **42**: 795–798.

27 Cole MW. Electronic surface states of liquid helium. *Rev Mod Phys* 1974; **46**: 451–464.
